# Genetic evolution of uveal melanoma guides the development of an inflammatory microenvironment

**DOI:** 10.1007/s00262-017-1991-1

**Published:** 2017-04-08

**Authors:** Gülçin Gezgin, Mehmet Dogrusöz, T. Huibertus van Essen, Wilhelmina G. M. Kroes, Gregorius P. M. Luyten, Pieter A. van der Velden, Vonn Walter, Robert M. Verdijk, Thorbald van Hall, Sjoerd H. van der Burg, Martine J. Jager

**Affiliations:** 1 Department of Ophthalmology, Leiden University Medical Center, PO Box 9600, 2300 RC Leiden, The Netherlands; 2 Department of Clinical Genetics, Leiden University Medical Center, Leiden, The Netherlands; 3 Department of Biochemistry, Penn State Milton S. Hershey Medical Center, Hershey, PA USA; 4 Department of Pathology, Section Ophthalmic Pathology, Erasmus MC University Medical Center, Rotterdam, The Netherlands; 5 Department of Clinical Oncology, Leiden University Medical Center, Leiden, The Netherlands

**Keywords:** BAP1, Infiltration, Macrophages, T cells, Lymphocytes, Chromosome

## Abstract

Uveal melanoma (UM) is characterized by a number of genetic aberrations that follow a certain chronology and are tightly linked to tumor recurrence and survival. Loss of chromosome 3, bi-allelic loss of BAP1 expression, and gain in chromosome 8q have been associated with metastasis formation and death, while loss of chromosome 3 has been associated with the influx of macrophages and T cells. We used a set of genetically-classified UM to study immune infiltration in the context of their genetic evolution. We show in two independent cohorts that lack of BAP1 expression is associated with an increased density of CD3^+^ T cells and CD8^+^ T cells. The presence of extra copies of chromosome 8q in disomy 3 tumors with a normal BAP1 expression is associated with an increased influx of macrophages (but not T cells). Therefore, we propose that the genetic evolution of UM is associated with changes in the inflammatory phenotype. Early changes resulting in gain of chromosome 8q may activate macrophage infiltration, while sequential loss of BAP1 expression seems to drive T cell infiltration in UM.

## Introduction

Uveal melanoma (UM) is the most common primary intraocular malignancy in Caucasian adults and may lead to metastatic disease in up to 50% of patients [1, 2]. Current treatments are hardly ever effective against metastases [3], and hence, most research efforts are focused on the development of targeted therapies or immunotherapeutic approaches, such as treatments with immune checkpoint inhibitors, vaccination, or adoptive T cell therapy [4–8].

Some UM express increased levels of human leukocyte antigen (HLA) class I and are infiltrated by macrophages and lymphocytes, and this is known as an inflammatory phenotype [9–12]. The presence of this inflammatory phenotype has been correlated with a specific genetic aberration, which is the loss of one copy of chromosome 3 (monosomy 3) [13]. Other chromosomal abnormalities frequently occur in chromosomes 1, 6, and 8 [14–17]. Following an initiating mutation in either GNAQ or GNA11, gain of 8q is thought to be one of the earliest genetic aberrations, followed by loss of one chromosome 3 [18, 19]. Gain of 8q and monosomy 3 and are both associated with the development of UM metastases and a poor prognosis [16, 20]. Similarly, gene expression analysis has been used to divide UM into two major classes, 1 and 2, which are good predictors of prognosis [21, 22]. Moreover, we recently showed that, in our hands, class II tumors can be subdivided into IIa and IIb: while class IIa tumors are composed of highly homogeneous tumor cells and class IIb tumors contain a larger percentage of non-tumor cells which are likely to be immune cells. Interestingly, class IIa and IIb tumors differed in their numbers of chromosome 8q copies [19].

Chromosome 3 contains the gene for *BRCA1-associated protein 1* (BAP1). In UM, inactivating hemizygous mutations in this gene have been found [23, 24], which are associated with loss of BAP1 protein expression and a high metastatic risk [23–26]. However, monosomy 3 and loss of BAP1 may occur independently, as tumors with a normal chromosome 3 status with lack of BAP1 expression have been identified, as well as tumors with monosomy 3 that still express BAP1. Such atypical tumors show high-risk clinico-pathological features and convey an increased metastatic risk [25]. BAP1 is a member of the ubiquitin-carboxy-terminal hydrolase (UCH) family [27], and is also known as ubiquitin carboxyl-terminal hydrolase L2 (UCHL2). Another member of the UCH family, ubiquitin carboxyl-terminal hydrolase L1 (UCHL1), is associated with suppressed production of pro-inflammatory chemokines and cytokines in keratinocytes [28]. We, therefore, hypothesized that loss of BAP1 expression might be related to macrophage and/or T cell infiltration.

To assess whether genetic alterations affect immune cell infiltration in UM, we studied the presence and type of tumor-infiltrating immune cells in UM subtypes consisting of typical cases of UM (e.g. disomy 3 tumors with BAP1 expression and monosomy 3 with loss of BAP1 expression) and atypical cases of UM (e.g. disomy 3 tumors with loss of BAP1 expression and monosomy 3 tumors with expression of BAP1). In addition, we studied disomy 3/BAP1-positive cases with and without extra copies of chromosome 8q.

Our data show that loss of BAP1 protein expression is predominantly related to T cell infiltration in UM, while early gain of chromosome 8q is associated with macrophage infiltration.

## Materials and methods

### Patient selection

Tumor tissue specimens were obtained from 84 UM patients. Patients underwent primary enucleation for UM between 1999 and 2008 at the Leiden University Medical Center (Leiden, The Netherlands). Part of the tumor was snap frozen using 2-methyl butane, and DNA and RNA were isolated. The remaining tumor was fixed in 4% neutral-buffered formalin for 48 h and subsequently embedded in paraffin. This study was approved by the Medical Ethics Committee of the Leiden University Medical Center. Tumor material was handled according to the Dutch National Ethical Guidelines (‘Code for Proper Secondary Use of Human Tissue’), and the tenets of the Declaration of Helsinki (World Medical Association of Declaration 2013; ethical principles for medical research involving human subjects). In addition, 80 patients from The Cancer Genome Atlas (TCGA) Project on UM were included in this study as an independent validation cohort.

### Immunohistochemistry and fluorescent immunostaining of the Leiden Cohort

4-µm serial sections from paraffin-embedded tissue were cut and used for immunostaining. Immunohistochemistry (IHC) of BAP1 was performed on 74 tumors, as described previously [24]. Tumors were scored by two independent investigators as BAP1-positive or -negative based on nuclear staining. Immunofluorescence (IF) staining for T cells and macrophages was performed on 43 tumors as described previously [29, 30]. T cell types were detected by primary antibodies: anti-CD3 (ab828, rabbit polyclonal; Abcam, Cambridge, MA, United States of America) and anti-CD8 (4B11, mouse monoclonal IgG2b; Novocastra, Valkenswaard, The Netherlands). To visualize the T cells, the following secondary antibodies were used: goat-anti-rabbit IgG Alexa 546 and goat-anti-mouse IgG2b Alexa 647 (Molecular Probes, Invitrogen, Breda, The Netherlands). Counts of intratumoral CD3^+^ and CD8^+^ T cells were represented as the number of cells per square millimeter. For IF staining of CD68^+^ macrophages, we used the primary mouse anti-human macrophage CD68 antibody (clone 514H12; ab49777; Abcam, Cambridge, United Kingdom), and as secondary antibody AlexaFluor IgG2a (488) goat-anti-mouse. The amount of CD68^+^ expression was determined in pixels per square millimeter.

### DNA and gene expression analysis

DNA and gene expression analysis were performed on 54 tumor specimens from Leiden, in which the BAP1 status was known. The QIAmp DNA Mini kit was used to isolate DNA for single-nucleotide polymorphism (SNP) analysis (Qiagen, Venlo, The Netherlands). SNP analysis was then performed with the Affymetrix 250K_NSP microarray and Affymetrix Cytoscan HD chip (Affymetrix, Santa Clara, California, United States of America) to detect aberrations of chromosome 3 as described previously [20]. Information on chromosome 8q was obtained by digital polymerase chain reaction (dPCR) [20]. A threshold of >2.1 was defined as having extra copies of chromosome 8q. The RNeasy mini kit was used to isolate RNA for gene expression analyses (Qiagen, Venlo, The Netherlands). Gene expression levels of *CD3* and *CD8* (T cells), *CD68* (macrophages), and pro-inflammatory cytokines, specifically *macrophage inflammatory protein 1α* (*CCL3*), *vascular endothelial growth factor A (VEGFA), stromal cell-derived factor 1 (CXCL12), CCL7, CSF-1, monocyte chemoattractant protein-1 (CCL2), RANTES (CCL5), interferon gamma-induced protein 10 (CXCL10), CCR7*, and *CXCR4* were obtained using the Illumina HT-12 v4 chip (Illumina, San Diego, California, United States of America). The pro-inflammatory cytokines were selected based on our previous papers [31–33]. We could validate the probes for *CD3,* *CD8* and *CD68* in 24 tumors in which gene expression levels had been determined with an Illumina HT12 v4 array and in which the number of infiltrating cells was analyzed by IF. In addition, data on RNA sequencing and Affymetrix SNP 6.0 array from 80 samples of UM were obtained from the TCGA Research Network: http://cancergenome.nih.gov/. Copy numbers for 8q were determined by Affymetrix SNP 6.0 array and analyzed with the GISTIC 2.0 algorithm [34, 35]. Copy numbers >2 were categorized as extra copies of chromosome 8q. *BAP1, CD68, CD3*, and *CD8* expression were obtained by RNA sequencing and quantified as log2(RSEM+1). *BAP1* expression was dichotomized into *BAP1*-positive and *BAP1*-negative expression at the median.

### Statistical analysis

Analyses were performed using SPSS version 20.0.0 (IBM SPSS Statistics, IBM Corporation, Armonk, New York, United States of America) and graphs were made using GraphPad Prism version 5.0 for Windows (GraphPad Software, La Jolla, California, United States of America, http://www.graphpad.com). The Mann–Whitney *U* test was applied for continuous parameters. Correlation analyses were performed with Spearman’s rho correlation test. A *P* value < 0.05 was considered statistically significant.

## Results

### Loss of BAP1-protein expression is associated with an inflammatory phenotype in UM

**Fig. 1:**
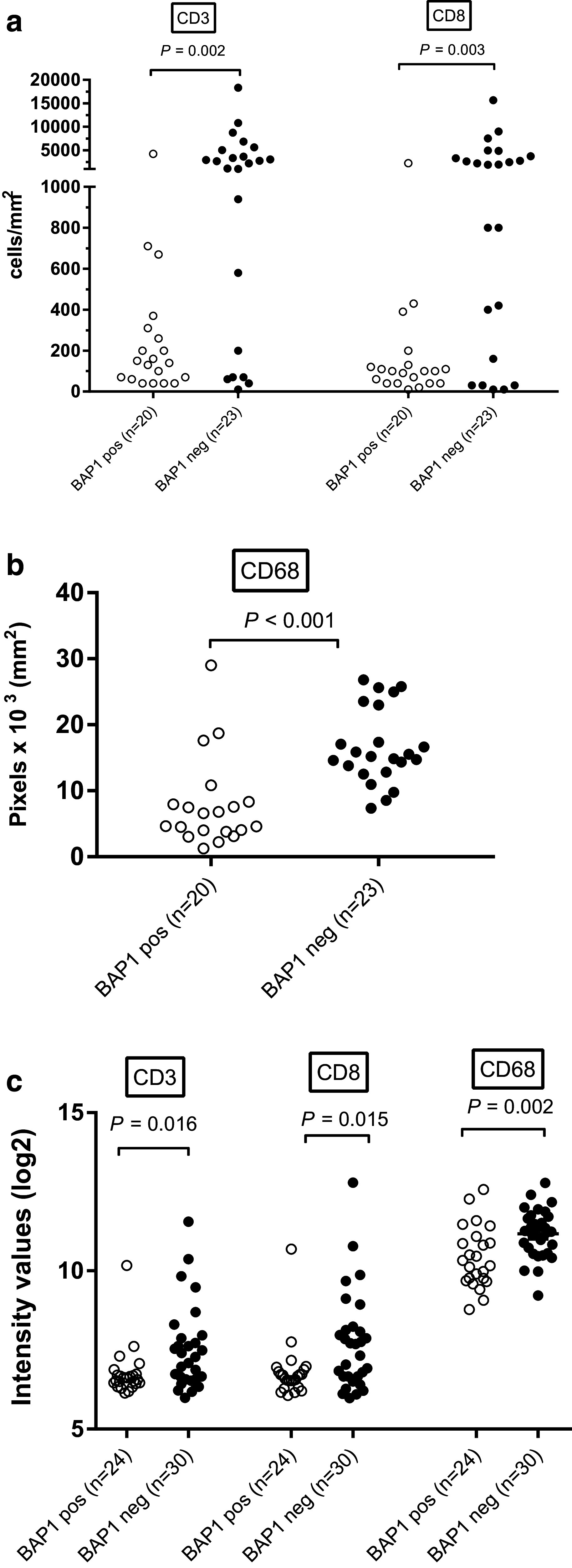
Presence of T cells (**a**), and macrophages (**b**) as determined by IF staining or by gene expression (**c**) was compared between BAP1-positive and BAP1-negative tumors from the Leiden cohort

As expression of UCHL1, a member of the UCH family, has been associated with suppression of pro-inflammatory cytokines and chemokines in keratinocytes [28], we determined whether loss of expression of another UCH-member, BAP1 might be correlated to the development of an inflammatory phenotype in UM. We quantified the number of tumor-infiltrating macrophages and T cells by immunofluorescence staining of tissue sections in a cohort of 20 BAP1-immunopositive tumors and 23 BAP1-immunonegative tumors from the Leiden cohort, and found that BAP1-negative tumors contained significantly higher numbers of CD3^+^ T cells (*P* = 0.002), CD8^+^ T cells (*P* = 0.003), and CD68^+^ macrophages (*P* < 0.001) (Fig. 1a-b).

To corroborate our findings, we analyzed gene expression array data from 54 cases of UM in which BAP1 expression as well as chromosome 3 and 8q status had been determined. We first correlated potentially-useful probes by comparing IF staining data for CD68^+^ macrophages, CD3^+^and CD8^+^ T cells with their corresponding CD68, CD3, and CD8 probes used for RNA gene expression analysis in a group of 24 patients for whom both tests were available. The probes with the highest correlation were used for further analyses (Table 1), since we assumed that these probes represent the corresponding protein expression most accurately. We subsequently compared the presence of specific probes with BAP1 expression: a higher expression of *CD3* (*P* = 0.016), *CD8* (*P* = 0.015), and *CD68* (*P* = 0.002) was observed in tumors that did not express BAP1 (*n* = 24) compared to tumors that did express BAP1 (*n* = 30; Fig. 1c), corroborating our results obtained with IF staining (Fig. 1a, b). These results indicate that loss of BAP1 expression in UM is associated with a higher T cell and macrophage infiltration.

**Table 1:** Correlation of the values of different probes obtained with an Illumina gene expression array with immunohistochemical data in the Leiden cohort

T cell type	Probe name	Probe number	R	*P* value
CD68 (*n* = 24)	**CD68 probe 1**	**ILMN_1714861**	**0.490**	**0.015**
	CD68 probe 2	ILMN_2359907	0.372	0.073
	CD68 probe 3	ILMN_2267914	0.252	0.235
CD3 (*n* = 24)	**CD3D probe 1**	**ILMN_2261416**	**0.760**	**<0.001**
	CD3D probe 2	ILMN_2325837	0.748	<0.001
	CD3E	ILMN_1739794	0.523	0.009
	CD3G	ILMN_1717197	0.622	0.001
CD8 (*n* = 24)	CD8A probe 1	ILMN_1768482	0.685	<0.001
	CD8A probe 2	ILMN_1760374	0.500	0.013
	**CD8A probe 3**	**ILMN_2353732**	**0.744**	**<0.001**
	CD8B probe 1	ILMN_1748601	0.126	0.558
	CD8B probe 2	ILMN_2354191	0.388	0.061

The highest correlated probes (in bold) were used for further analysis

*P* values were obtained by the Spearman’s Rho correlation test

*n* number of patients, *R* correlation coefficient

**Fig. 2:**
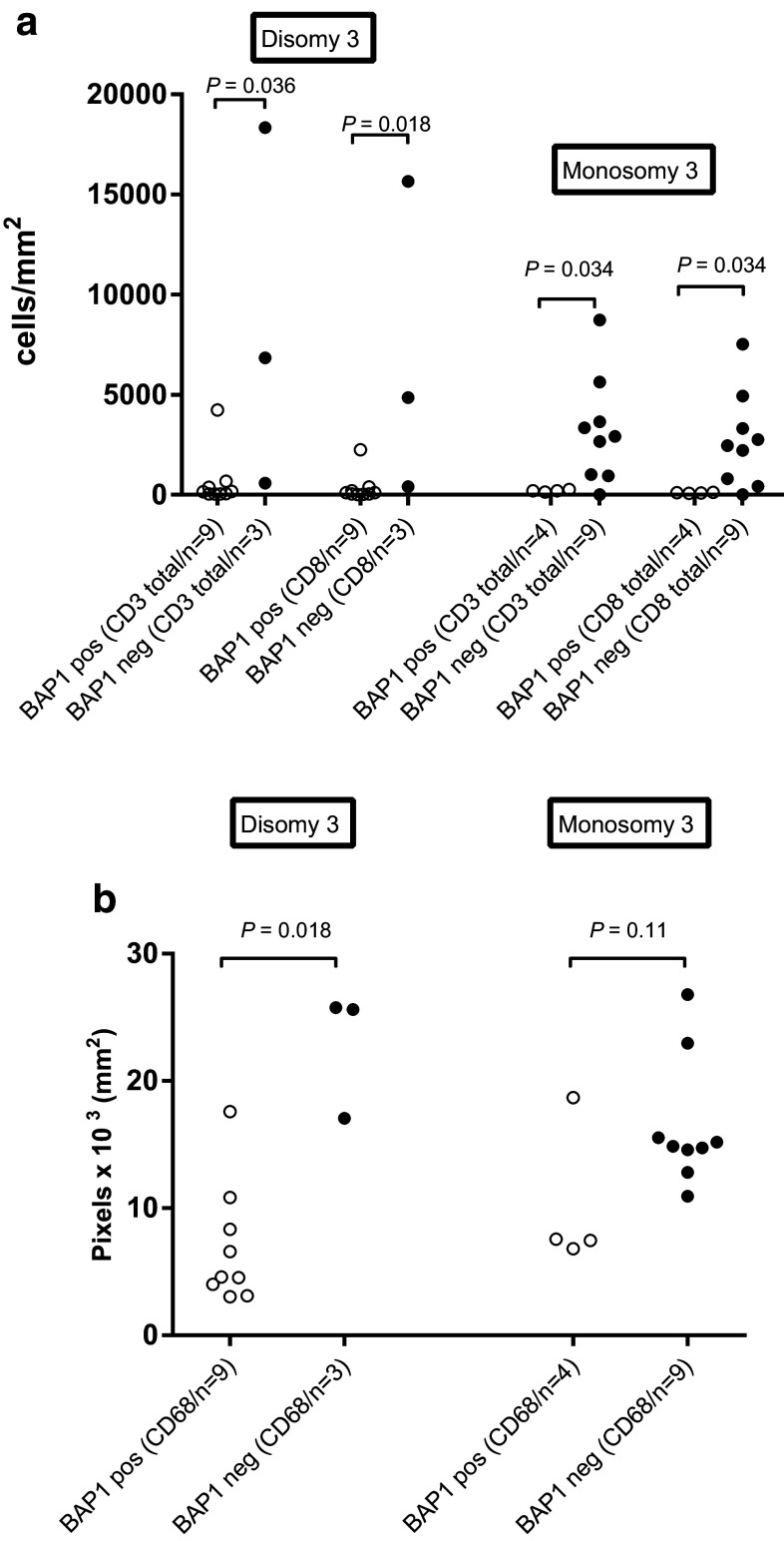
Comparison of T cells (**a**) and macrophages (**b**) as determined by IF staining between BAP1-positive and BAP1-negative tumors from the Leiden cohort that were either disomic or monosomic for chromosome 3 according to the Mann–Whitney *U* test

Since most monosomy 3 tumors lack BAP1 expression, it is difficult to determine whether the effects observed are due to loss of BAP1 expression or due to loss of one chromosome 3. We therefore, focused on atypical cases, and observed that within the disomy 3 tumors, loss of BAP1 protein expression was associated with increased numbers of CD3^+^ T cells (*P* = 0.036), CD8^+^ T cells (*P* = 0.018) and CD68^+^ macrophages (*P* = 0.018). Within the monosomy 3 tumors, we noticed that the expression of BAP1 protein was associated with significantly lower numbers of CD3^+^ T cells (*P* = 0.034) and CD8^+^ T cells (*P* = 0.034), but not of CD68^+^ macrophages (*P* = 0.11; Fig. 2a-b). This shows that in disomy 3 as well as monosomy 3 tumors, the loss of BAP1 protein is associated with more infiltrating T cells.

**Fig. 3:**
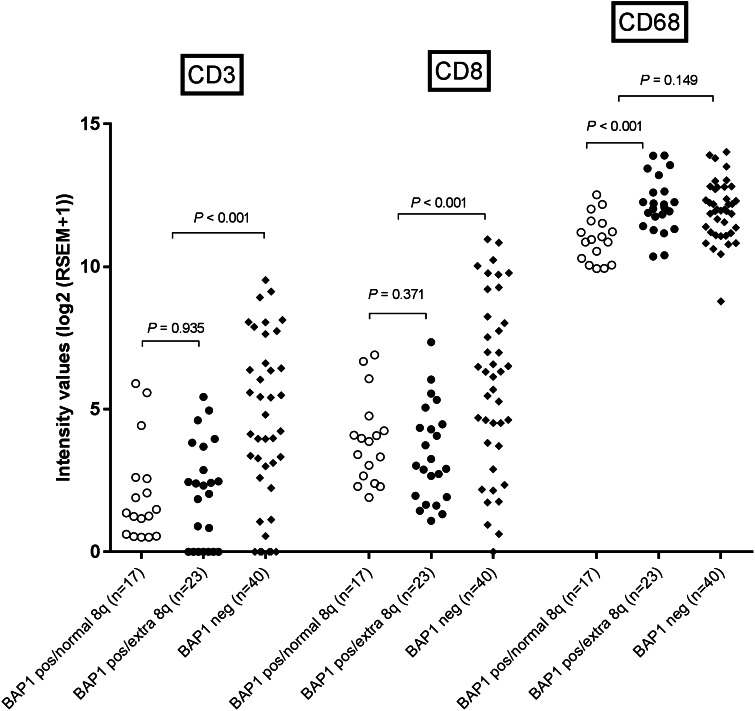
Comparison of T cells and macrophages as determined by gene expression between tumors from the TCGA cohort with a normal and abnormal chromosome 8q status in cases that had low (*BAP1*-negative) or high (*BAP1*-positive) *BAP1* expression as determined by RNA sequencing

In addition, we scrutinized an independent cohort that was available from the TCGA for a potential association between the BAP1-status and infiltration with T cells and macrophages. *BAP1*-negative tumors showed a higher expression of *CD3* (*P* < 0.001) and *CD8* (*P* < 0.001), but not for *CD68* (*P* = 0.149) than *BAP1*-positive tumors (Fig. 3), which confirms our finding that loss of BAP1 is associated with a higher density of T cells in UM.

Immune cell infiltration of tumors is governed by the local production of cytokines and chemokines. We analyzed whether the expression of several cytokines and chemokines involved in T cell and macrophage chemotaxis differed between 24 BAP1-positive and 30 BAP1-negative tumors from the Leiden cohort [31, 32]. Gene expression of *CCL5* and *CXCL10*, important chemokines in T cell chemotaxis, was higher in the BAP1-negative tumors (*P* < 0.001; *P* = 0.005 and *P* = 0.065; Table 2a).

**Table 2:** Expression of pro-inflammatory chemokines and receptors in BAP1-positive and BAP1-negative UM (**a**), and in disomy 3/BAP1-positive tumors, with and without 8q gain (**b**) in the Leiden cohort

(a)	BAP1-positive (*n* = 24)	BAP1-negative (*n* = 30)	*P* value^+^
CCL3 (MIP-1α)	6.7 (6.5–8.5)	6.7 (6.3–8.0)	0.321
VEGFA probe 1	6.8 (6.5–7.4)	6.8 (6.5–7.3)	0.651
VEFGA probe 2	6.4 (6.3–6.8)	6.4 (6.2–6.8)	0.403
CXCL12 (SDF-1) probe 1	7.1 (6.6–8.6)	7.3 (6.5–9.4)	0.689
CXCL12 (SDF-1) probe 2	6.6 (6.3–7.1)	6.6 (6.3–7.6)	0.175
CXCL12 (SDF-1) probe 3	6.5 (6.2–7.1)	6.5 (6.0–7.3)	0.848
CCL7	6.2 (6.0–6.5)	6.3 (5.9–6.7)	0.088
CSF-1	6.4 (6.3–6.7)	6.5 (6.3–6.7)	0.130
CCL2 (MCP-1)	7.2 (6.4–9.5)	7.1 (6.4–9.1)	0.614
CCL5 (RANTES) probe 1	6.9 (6.4–10.7)	7.5 (6.5–12.1)	<**0.001**
CCL5 (RANTES) probe 2	7.7 (6.6–12.4)	8.8 (7.0–14.4)	**0.005**
CXCL10 (IP-10)	6.8 (6.4–10.5)	7.4 (6.4–10.4)	0.065
CCR7	6.4 (6.1–7.0)	6.3 (6.0–7.2)	0.903
CXCR4 probe 1	6.5 (6.2–7.2)	6.7 (6.2–8.3)	**0.038**
CXCR4 probe 2	6.5 (6.1–6.9)	6.4 (6.2–6.8)	**0.010**
CXCR4 probe 3	6.6 (6.2–7.5)	6.8 (6.2–8.2)	0.216

(b)	D3/BAP1+/n8q (*n* = 9)	D3/BAP1+/8q gain (*n* = 8)	*P* value*
CCL3 (MIP-1α)	6.6 (6.5–6.8)	6.8 (6.7–8.1)	**0.002**
VEGFA probe 1	6.7 (6.5–6.9)	6.9 (6.6–7.4)	0.059
VEFGA probe 2	6.4 (6.3–6.6)	6.5 (6.3–6.8)	0.236
CXCL12 (SDF-1) probe 1	7.1 (6.7–7.9)	7.1 (6.6–8.6)	0.743
CXCL12 (SDF-1) probe 2	6.5 (6.3–6.8)	6.5 (6.4–6.8)	0.743
CXCL12 (SDF-1) probe 3	6.5 (6.3–6.7)	6.5 (6.2–7.0)	0.888
CCL7	6.1 (6.0–6.3)	6.3 (6.1–6.4)	0.277
CSF-1	6.4 (6.3–6.6)	6.4 (6.3–6.6)	0.606
CCL2 (MCP-1)	6.8 (6.4–7.7)	7.3 (6.8–9.1)	0.059
CCL5 (RANTES) probe 1	6.8 (6.4–7.0)	6.9 (6.5–7.5)	0.236
CCL5 (RANTES) probe 2	7.4 (6.6–8.2)	7.9 (7.0–8.9)	0.277
CXCL10 (IP-10)	6.7 (6.4–7.4)	6.8 (6.5–8.4)	0.321
CCR7	6.3 (6.1–6.6)	6.4 (6.2–6.7)	0.606
CXCR4 probe 1	6.4 (6.2–6.7)	6.5 (6.3–6.9)	0.114
CXCR4 probe 2	6.5 (6.1–6.6)	6.5 (6.2–6.9)	0.370
CXCR4 probe 3	6.5 (6.2–6.9)	6.6 (6.3–7.3)	0.321

The boldface indicates a significant difference

*D3* disomy 3, *BAP1+* positive BAP1 protein expression, *n8q* normal chromosome 8q status, *8q gain* chromosome 8q gain, *n* number of patients; median (range)

^+^ *P* value comparison of chemokine expression in BAP1-positive and BAP1-negative tumors

*P value comparison of chemokine expression in tumors with D3/BAP1+/n8q and tumors with D3/BAP1+/8q gain

Altogether, these results show that the production of immune cell-attracting chemokines and infiltration of different types of immune cells in UM are associated with the loss of BAP1 expression, independent of chromosome 3 loss.

### Influence of chromosome 8q gain on the inflammatory microenvironment

**Fig. 4:**
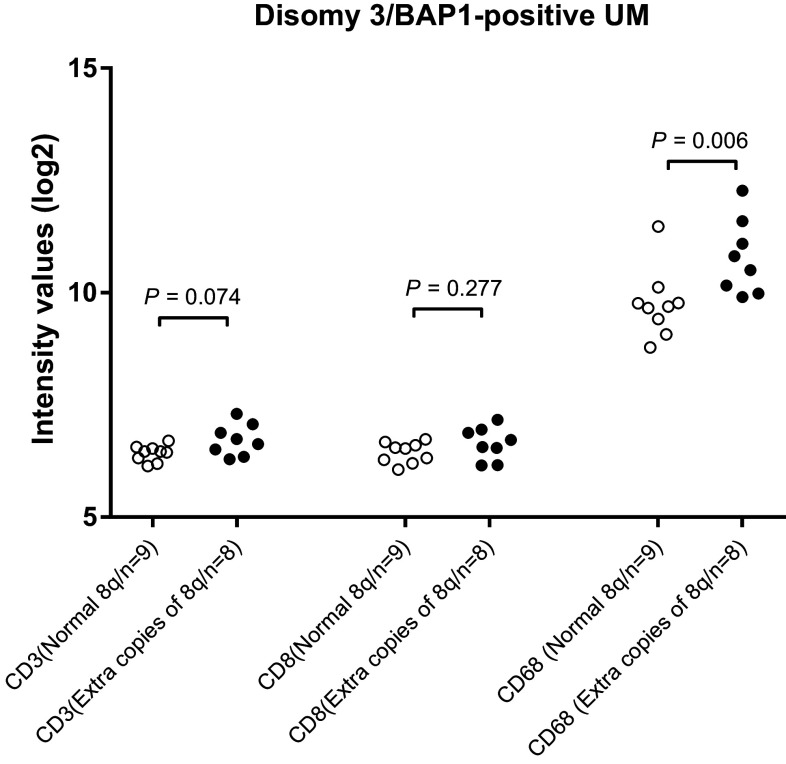
Comparison of T cells and macrophages as determined by gene expression between tumors from the Leiden cohort with a normal and abnormal chromosome 8q status in cases that were disomic for chromosome 3 and BAP1-positive as determined by IHC staining

Gain in the copy number of chromosome 8q is considered an early event in UM development, occurring before loss of chromosome 3 [18, 19]. Seventeen cases of UM from Leiden were available in which the tumor showed gain in chromosome 8q but did not show loss of chromosome 3 or lack of BAP1 protein expression. These cases allowed us to study the influence of gain of chromosome 8q on immune cell infiltration. Tumors that carried additional copies of chromosome 8q had an increased expression of *CD68* (*P* = 0.006), but not of *CD3* and *CD8* (Fig. 4). Again, using the TCGA data as an independent cohort, we analyzed whether there was an association between gain of chromosome 8q and the presence of macrophages and T cells. *BAP1-*positive tumors with extra copies of 8q had an increased expression of *CD68* (*P* < 0.001) (Fig. 3) but not of *CD3* or *CD8* compared to *BAP1*-positive tumors with a normal chromosome 8q. Since these tumors had no loss of chromosome 3 or aberrant expression of BAP1, these data confirm our finding in the Leiden cohort that the early gain in chromosome 8q is responsible for macrophage infiltration but not for T cell infiltration.

In addition, we determined whether, in the disomy3/BAP1-positive tumors from the Leiden cohort, extra copies of chromosome 8q affect the expression of pro-inflammatory cytokines. The myeloid-cell attracting chemokines *CCL3* and *CCL2* showed a higher expression in disomy3/BAP1-positive tumors (*P* = 0.002 and *P* = 0.059, respectively) that carried extra copies of chromosome 8q than did disomy3/BAP1-positive tumors with two copies of chromosome 8q (Table 2b). This was not the case for the expression of typical T cell attracting chemokines, such as CCL5, CXCL12, and CXCL10.

## Discussion

We show that the presence of extra copies of chromosome 8q in UM is associated with macrophage infiltration, while loss of BAP1 protein expression, with or without loss of chromosome 3, is associated with T cell infiltration in UM.

The chromosomal evolution of aggressive UM is thought to start with a mutation in GNAQ/GNA11 [36, 37], followed by gain of chromosome 8q that precedes a potential loss of one copy of chromosome 3 and/or mutation in the *BAP1* gene [20, 38]. We show that in UM with disomy 3 and expression of BAP1, the presence of additional copies of chromosome 8q is highly associated with the increased expression of macrophage-attracting chemokines and a stronger macrophage infiltration. In this subgroup, no effect was found with respect to the production of chemokines associated with T cell infiltration. This phenomenon could not be assessed in monosomy 3 tumors as more than 90% of monosomy 3 UM carry extra copies of 8q. Thus, a gain in copy number of chromosome 8q is associated with an increase in macrophage infiltration.

One might expect that this influx is initiated by activation of the c-Myc gene, a proto-oncogene located on chromosome 8q24, which is upregulated in many types of cancer and has been studied in UM [18, 39, 40]. It has previously been suggested that c-Myc may be involved in the activation of inflammatory mediators in the tumor microenvironment [41]. However, we previously observed the opposite, i.e., an association between a high c-Myc expression and a low inflammatory phenotype, making it unlikely that c-Myc is the relevant factor [42].

Monosomy 3 and loss of BAP1-protein expression are strongly correlated in UM, but we observed several atypical cases which allowed us to separately assess the contribution of chromosome 3 and BAP1-protein expression on the magnitude and type of immune cell infiltration and to pinpoint that it was the loss of BAP1 which was associated with the higher expression of T cell-attracting chemokines and a stronger T cell infiltration in UM. The previous studies have reported that tumor suppressor proteins can be involved in the processes and pathways of tumor-promoting inflammation by interacting with transcription factors such as nuclear factor-κB (NF-κB) [43]. NF-κB regulates genes which are involved in inflammation and immune responses. A close family member of BAP1 is UCHL1. Similar to BAP1, UCHL1 functions as a tumor suppressor protein [44] and was recently shown to suppress the NF-κB pathway, thereby negatively affecting the production of type 1 interferon and pro-inflammatory cytokines and chemokines, including CCL5 [28]. We, therefore, hypothesize that BAP1 may have a similar function as UCHL1 and that loss of BAP1 alleviates the suppression pathways leading to activation of NF-κB, resulting in the production of cytokines and chemokines that attract tumor-specific T cells into UM.

Obvious correlations between genetic changes and the development of an immune infiltrate are not easy to find. Loss of function of several tumor suppressor genes (*p53, PTEN*) due to genetic aberrations is known to be associated with inflammation [45]. Interestingly, loss of BAP1 in an unusual cutaneous tumor, the atypical Spitz nevus, was associated with a higher presence of T cells [46].

When looking at UM, one of the chemokines that was higher in BAP1-negative than BAP1-positive tumors was *CCL5*; another chemokine that was almost significantly higher in BAP1-negative tumors was CXCL10. CCL5 and CXCL10 play a role in the recruitment of T cells [47]. An influence of BAP1 on NF-κB and the additional release of pro-inflammatory multifunctional chemokines might explain why both macrophages and T cells are found in BAP1-negative UM, but this requires further research.

Another chemokine involved in T cell recruitment is CXCL12, which is the ligand for chemokine receptor CXCR4. Previously, it has been described that CXCR4 is involved in the migration of UM cells to the liver [48, 49]. In contrast, another group reported that the expression of CXCR4 was, indeed, correlated with lymphocyte infiltration, but had no prognostic relevance in UM patients [33]. We observed discrepant results for CXCR4 in our Leiden cohort, with one probe showing a higher expression in BAP1-negative tumors, and another one a lower expression (Table 2a).

Previously, it had been shown that tumor-intrinsic active β-catenin signaling restrains tumor-infiltration by T cells, resulting in the escape of tumors from immune surveillance [50]. The β-catenin protein is encoded by the gene *CTNNB1*, which, like *BAP1*, is located on chromosome 3p21. Hence, loss of chromosome 3 may also reduce β-catenin expression. In contrast to *BAP1* [24, 25], however, there is no evidence that the other allele of *CTNNB1* is frequently mutated in UM and thus that the loss of β-catenin signalling is underlying T cell infiltration in UM. In our cohort, we found no correlation between *CTNNB1* expression and the amount of CD8^+^ T cells. Furthermore, the number of β-catenin-positively staining tumor cells in UM is around 10% [51], making its expression an unlikely explanation for the absence of T cell infiltration in most UM.

Our current findings show that alterations in copy numbers or mutations in certain genes can drive a specific type of immune response. As the most common treatment of UM is irradiation and not enucleation, we wondered whether local treatments might affect immune infiltration in UM. No tumors in either the Leiden cohort or the TCGA cohort had received prior irradiation. A previous study from our group showed that more T cells were present in secondarily enucleated eyes after prior irradiation compared to primarily enucleated eyes [52]. As irradiation influenced our chromosome testing, we could not always analyze the chromosome status in previously irradiated tumors [53]. We do not yet know how the type of inflammation or irradiation influences the patient’s response to immunotherapy, which at this moment has not been very successful in UM.

In conclusion, we provide evidence that the magnitude and type of immune cell infiltration observed in the subgroup of inflamed UM co-evolve with the sequential genetic changes occurring in UM. The initial infiltration by macrophages is related to a gain in the copy number of chromosome 8q, while additional T cell infiltration is correlated to a loss of functional BAP1-protein expression.

## Abbreviations

*BAP1*: BRCA1-associated protein 1
*CCL*2: Chemokine (C-C motif) ligand 2 (Monocyte chemoattractant protein-1)
*CCL3*: Chemokine (C-C motif) ligand 3 (Macrophage inflammatory protein 1α)
*CCL5*: Chemokine (C-C motif) ligand 5 (RANTES)
*CXCL10*: Chemokine (C-X-C motif) ligand 10 (Interferon gamma-induced protein 10)
*CXCL12*: Chemokine (C-X-C motif) ligand 12 (Stromal cell-derived factor 1)
*dPCR*: Digital polymerase chain reaction
*HLA*: Human leukocyte antigen
*IF*: Immunofluorescence
*IHC*: Immunohistochemistry
*NF-κB*: Nuclear factor κB
*SNP*: Single-nucleotide polymorphism
*TCGA*: The Cancer Genome Atlas
*UCH*: Ubiquitin-carboxy-terminal hydrolase
*UCHL1*: Ubiquitin carboxyl-terminal hydrolase L1
*UCHL2*: Ubiquitin carboxyl-terminal hydrolase L2
*UM*: Uveal melanoma
*VEGFA*: Vascular Endothelial Growth Factor A
